# Optogenetic dissection of basolateral amygdala projections during cue-induced reinstatement of cocaine seeking

**DOI:** 10.3389/fnbeh.2013.00213

**Published:** 2013-12-24

**Authors:** Michael T. Stefanik, Peter W. Kalivas

**Affiliations:** Department of Neuroscience, Medical University of South CarolinaCharleston, SC, USA

**Keywords:** optogenetics, prelimbic cortex, nucleus accumbens, cocaine, reinstatement

## Abstract

Stimuli previously associated with drugs of abuse can become triggers that elicit craving and lead to drug-seeking behavior. The basolateral amygdala (BLA) is a key neural structure involved in cue-induced reinstatement of cocaine seeking. Previous studies have also implicated projections from the BLA directly to the nucleus accumbens (NAc) in these behaviors. However, other structures critically involved in cocaine seeking are targets of BLA innervation, including the prelimbic prefrontal cortex (PL). It has been shown that BLA or PL innervation direct to the NAc can modulate reward-related behaviors but the BLA also projects to the PL, and given the importance of the PL projection to the NAc for reinstated drug seeking, we hypothesized the BLA to PL projection may indirectly influence behavior via PL innervation to the NAc. We delivered a virus expressing the inhibitory optogenetic construct ArchT into the BLA and implanted fiber optics above the injection site or axon terminal fields in either the NAc or PL. Rats then went through 12 days of cocaine self-administration followed by extinction training. Following extinction, animals underwent cue-induced reinstatement sessions in the presence or absence of optical inhibition. Inactivation of the BLA and either the BLA core subcompartment of the NAc (BLA-to-NAcore) BLA-to-PL projections inhibited cue-induced reinstatement. These data demonstrate that the BLA projection either directly into the NAc, or indirectly via the PL, is a necessary regulator of drug-seeking behavior.

## Introduction

Exposure to drug-associated cues elicits craving and increases the probability that drug users will relapse, even after extended periods of abstinence. Understanding the neural circuits that underlie relapse is imperative in order to identify targets for therapeutic intervention. A dynamic interaction between basolateral amygdala (BLA) and prefrontal cortex (PFC) inputs to the nucleus accumbens (NAc) is part of the neural circuitry underpinning cue-induced reinstatement of drug seeking. Excitatory inputs carrying goal- and reward-related information from both cortical and limbic structures converge on the NAc, are integrated, and ultimately influence reward-directed actions. Encoding of reward-predictive stimuli by the NAc relies on synaptic activity from both the BLA and prelimbic region (PL; McGinty and Grace, 2008). The BLA is critical for generating a response to conditioned cues (Buffalari and See, 2010), and direct innervation of the NAc by the BLA is necessary for cue-induced reward seeking (Setlow et al., 2002; Di Ciano and Everitt, 2004; Ambroggi et al., 2008; Mashhoon et al., 2010; Shiflett and Balleine, 2010; Stuber et al., 2011). However, these functional studies have assessed only one projection or relied on unilateral disconnections between the two regions. This leaves open the possibility that a BLA-core subcompartment of the NAc (NAcore) disconnection could impair behavior by nonspecifically interrupting other pathways, including a projection from the PL of the PFC that receives BLA afferents and in turn projects to the NAcore. Consistent with this hypothesis, previous work has demonstrated BLA control over NAc activity depends on an interaction with the PFC (Jackson and Moghaddam, 2001; McGinty and Grace, 2008), and that this interaction plays a role in cue-induced reinstatement (Fuchs et al., 2007; Mashhoon et al., 2010). Similarly, PFC neurons projecting to the NAc are also excited by conditioned stimuli (Ishikawa et al., 2008; McGinty and Grace, 2008), are essential for reward-seeking behavior (Park et al., 2002; McFarland et al., 2003; Stefanik et al., 2013b), and it has been hypothesized maladaptive changes in this pathway may be a common neural substrate that underlies the unmanageable drive to seek drugs (Goldstein and Volkow, 2002; Kalivas and Volkow, 2005).

Given that the BLA also projects to the PL (Sarter and Markowitsch, 1983; Reep, 1984), and that BLA inactivation can influence NAc projecting neurons from the PL (Jackson and Moghaddam, 2001; McGinty and Grace, 2008), it seems possible that this indirect pathway to the NAc might be an additional route in which BLA activity is influencing cued reinstatement. To test this hypothesis, we employed an inhibitory optogenetic strategy in which we delivered an adeno-associated virus (AAV) coding for the light sensitive proton pump archaerhodopsin (ArchT; Chow et al., 2010) into the BLA and selectively inhibited axon terminals in either the NAc or PL during cue-induced reinstatement of cocaine seeking.

## Materials and methods

### Animal housing and surgery

All methods used were in compliance with the National Institutes of Health *Guide for the Care and Use of Laboratory Animals* and were approved by the Medical University of South Carolina’s Institutional Animal Care and Use Committee. Male Sprague Dawley rats (250–300g, Charles River Laboratories) were individually housed under temperature- and humidity-controlled conditions with a 12 h reverse light/dark cycle (lights on at 6:00 P.M.). Rats were fed *ad libitum* until 7 days post-surgery, after which food was restricted to 25 g of chow pellets per day.

Following one week of handling and acclimation, rats underwent surgery for injection of AAV, implantation of fiber optics, and implantation of indwelling jugular catheters. Animals were anesthetized with ketamine HCl (87.5 mg/kg, i.m.) and xylazine (5 mg/kg, i.m.). Ketorolac (3mg/kg, i.p.) was administered before surgery to provide analgesia. Intra-jugular catheters were implanted as previously described (LaLumiere et al., 2012). Catheters were flushed daily with cefazolin (0.2 mL of 0.1 g/mL) and heparin (0.2 mL of 100 IU) for 1 week, then daily with heparin for the remainder of the experiment to maintain catheter patency.

For virus injections, 0.7 μl of virus (rAAV2-CAG-ArchT-GFP or rAAV2-CMV-GFP for BLA cell body experiment, ∼10^12^ viral particles/ml) was delivered bilaterally through 33 gauge needles (0.14 μL/min for 5 min). Needles were left in place for 10 min following injection to allow for virus diffusion away from injection site. For the virus injections, coordinates from Bregma were as follows: BLA: −2.8 mm anteroposterior, ±5.0 mm mediolateral, −8.5 mm dorsoventral. For fiber optic implantation, chronically implantable fiber optics (Precision Fiber) were implanted 0.5 mm dorsal to the site intended to receive light stimulation, coordinates from Bregma: NAcore: +1.5 mm anteroposterior, +3.5 mm mediolateral, −6.5 dorsoventral (10° angle); PL: +3.1 mm anteroposterior, +2.0 mm mediolateral, −4.0 dorsoventral (12° angle). Fibers we secured to the skull using small screws and dental acrylic and animals recovered for 1 week before behavioral testing.

### Self-administration, extinction and reinstatement procedures

Self-administration, extinction, and reinstatement procedures occurred in standard operant chambers equipped with two retractable levers, a house light, cue light, and 2900 Hz tone generator (Med Associates). Before cocaine self-administration training, animals were food deprived for 24 h and then underwent a single 15 h food training session in which presses on the active lever resulted in the delivery of a single food pellet (45mg, Noyes) on a fixed-ratio 1 (FR1) schedule of reinforcement. Following food training, animals were restricted to 25 g of food per day, given immediately after the behavioral session, for the remainder of the experiment. One day later, animals began 2-h sessions cocaine self-administration on an FR1 schedule with a 20 s time out. Each active lever press resulted in a 0.05 ml infusion of 0.20 mg cocaine (∼15–20 mg/kg per animal total over 2-h session, dissolved in 0.9% sterile saline, NIDA) and the drug-paired cues (concurrent illumination of the stimulus light above the active lever and tone) for 5 s. Active lever presses made during the time out were counted but did not result in drug delivery and inactive lever presses were of no consequence. Rats underwent self-administration 6 days/week for at least 2 weeks (minimum of 12 days), until they met maintenance criteria of ≥10 infusions of cocaine over 10 days, as well as discrimination between active and inactive levers (>75% lever presses on active lever). A total of three rats not reaching these criteria after 4 weeks were excluded from the study.

Following successful acquisition and maintenance of cocaine self-administration, extinction training (2 h/day) began. During extinction, presses on the previously active lever were recorded but no longer produced drug or presentation of the drug-paired cues. All rats underwent at least 10 days of extinction, until active lever pressing fell to <30% of the average responding during self-administration. Animals were habituated to the fiber optic leashes for ≥3 sessions of both self-administration and extinction. Immediately before testing, fibers were attached and remained in place for the duration of the session. During the reinstatement sessions, active lever presses produced the light/tone drug-paired cues that had been presented during self-administration, but no drug was delivered. All animals underwent two reinstatement sessions, counterbalanced with respect to whether illumination was given.

### Optical inhibition

Optical inhibition was delivered as previously described (Stefanik et al., 2013a,b). Briefly, chronically implantable optical fibers were housed inside stainless steel ferrules (for construction details, see Sparta et al., 2011) and implanted bilaterally ∼0.5 mm dorsal to the site intended to receive light. To permit bilateral inhibition, the single end of 2 × 1 fiber splitter (Precision Fiber) was connected via FC/PC connection to a rotating optical commutator, which was then attached via a fiber to a laser (diode-pumped solid-state, 200 mW, 561 nm multimode FC/PC fiber coupler connection, OEM Laser Systems). The two split ends of the fiber splitter were threaded through a metal leash, and epoxied into stainless steel ferrules which were then connected to the animal’s head via ceramic sleeves. Light output was measured with an optical power meter and adjusted to ∼10 mW of 561 nm light. Based on *in vivo* measurements, of light output in mammalian brain tissue, these parameters would be expected to provide sufficient light to at least 0.4 mm^3^ of tissue (Yizhar et al., 2011). Light was applied continuously for the 2 h reinstatement session, a procedure previously shown to inhibit neuronal firing without significantly desensitizing the opsin (Huff et al., 2013; Stefanik et al., 2013a,b; Tsunematsu et al., 2013). As work in our lab has previously shown no effect of light delivery to the NAcore or PL on behavior, GFP control experiments were conducted at the BLA cell bodies to control laser light on cue-induced reinstatement (Stefanik et al., 2013a,b).

### Immunohistochemistry and imaging

For immunohistochemistry and imaging, animals were anesthetized with pentobarbital (100 mg/ml, i.p.) and then transcardially perfused with phosphate-buffered saline (PBS, pH 7.4) followed by PBS containing 4% (w/v) paraformaldehyde. Brains were post-fixed for 24 h at room temperature in the perfusion solution. Coronal sections (75 μm thick) were incubated for 60 min in 1% hydrogen peroxide, rinsed three times in PBS, and then incubated overnight in PBS containing 0.25% triton-X, 0.01% sodium azide, and anti-GFP (rabbit, 1:50,000, Abcam) antibody. Sections were then rinsed once in PBS and incubated for 30 min in PBS containing the biotinylated secondary antibody (donkey, 1:1000, Jackson Immunoresearch) for 30 min, rinsed four times in PBS, and incubated for 1 h in an ABC Kit (Vector Labs). Sections were then rinsed once in PBS and incubated in PBS with 0.05% diaminobenzidine with 0.05% hydrogen peroxide for 5 min. Slices were then mounted and ArchT-expression was visualized on a light microscope.

### Data analysis

Statistics were performed using Prism (GraphPad Software). Reinstatement sessions were compared between extinction pressing, cue-induced reinstatement without laser, and cue-induced reinstatement with laser, using a two-tailed paired Student’s *t*-test with probability values adjusted according to the method of Bonferroni. Data are presented as mean ± SEM.

## Results

### Optical inhibition of the basolateral amygdala (BLA) reduces cue-induced reinstatement

**Figure 1:**
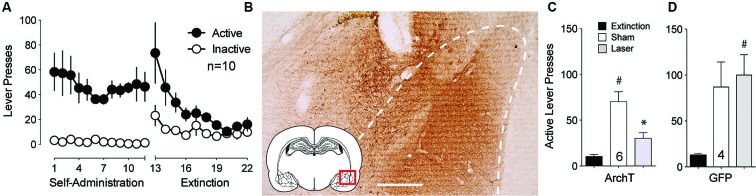
**Inhibition of BLA cell bodies reduces cue-primed reinstatement of cocaine seeking**. **(A)** Active lever pressing during self-administration and extinction training for this group of rats (*n* = 10). **(B)** DAB staining shows ArchT-expressing neurons in the BLA. Dashed line outlines BLA. Scale bar, 300 μm. **(C)** Inactivation of ArchT-expressing neurons in the BLA significantly reduces cue-primed reinstatement of cocaine seeking. **(D)** Illumination of control GFP virus has no effect on reinstated cocaine seeking. ^#^*p* < 0.05 compared with extinction levels of lever pressing for ArchT-expressing animals using a paired Student’s *t*-test with probability values adjusted for multiple comparisons according to Bonferroni, Sham *t*(5) = 5.50, *p* < 0.01; Laser *t*(5) = 4.15, *p* < 0.01. *p < 0.05 comparing laser and sham treatments, *t*(5) = 3.99, *p* = 0.010. For GFP controls, Sham *t*(3) = 2.91, *p* = 0.06; Laser *t*(3) = 4.32, *p* = 0.02. Comparing laser and sham treatments, *t*(3) = 0.77, *p* = 0.50.

The BLA is critically involved in the processing of cue-related information (Buffalari and See, 2010). To examine whether optical inhibition of this structure could alter cue-induced reinstated cocaine seeking, an AAV expressing the light-activated inhibitory proton pump ArchT (Chow et al., 2010) was microinjected into the BLA and fiber optics were implanted ∼0.5 mm dorsal to the injection site. Following self-administration and extinction training (Figure 1A), animals underwent cue-induced reinstatement in the presence or absence of ∼10 mW of 561 nm light in the BLA using a counterbalanced, within-subjects design. Rats microinjected with a control AAV virus expressing only GFP were treated identically. Figure 1B shows DAB staining for ArchT-GFP expression in the BLA. ArchT-expressing animals receiving laser light showed a reduction to nearly extinction levels of active lever-pressing compared to sham (no laser delivery) treatment (Figure 1C). To control for the potential effects of light delivery to this region, we showed that animals receiving the control GFP virus showed no difference in lever pressing regardless of the presence or absence of laser light (Figure 1D).

### Inhibiting the basolateral amygdala (BLA) to core subcompartment of the NAc (NAcore) projection inhibits cue-induced reinstatement



**Figure 2:**
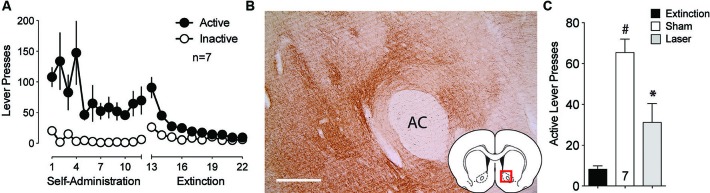
**Silencing of BLA-to-NAcore projections inhibit cue-primed reinstatement. (A)** Active lever pressing during self-administration and extinction training (*n* = 7). **(B**) ArchT-expressing terminal fibers in the NAcore after virus injection in the BLA. Scale bar, 300 μm. **(C)** Inactivation of ArchT-expressing fibers in the NAcore significantly reduces cue-primed reinstatement of cocaine seeking. ^#^*p* < 0.05 compared with extinction levels of lever pressing, using a paired Student’s *t*-test with probability values adjusted for multiple comparisons according to Bonferroni, Sham *t*(6) = 8.19, *p* < 0.001; Laser *t*(6) = 2.38, *p* = 0.110. *p < 0.05 comparing laser and sham treatments, *t*(6) = 3.54, *p* = 0.024.

Multiple studies point to the projection from the BLA to the NAcore as being critically involved cue-induced reward seeking (Setlow et al., 2002; Di Ciano and Everitt, 2004; Ambroggi et al., 2008; Mashhoon et al., 2010; Shiflett and Balleine, 2010; Stuber et al., 2011). To assess the involvement of this connection in cue-induced reinstated cocaine seeking, ArchT was microinjected into the BLA and optic fibers were implanted above terminal regions in the NAcore. Animals underwent self-administration and extinction training (Figure 2A). DAB staining for ArchT-GFP confirmed strong expression of the virus at the terminal fields in the NAcore (Figure 2B). Figure 2C shows significant reduction in active lever pressing produced by optical inhibition compared to sham treatment.

### Inhibiting the basolateral amygdala (BLA) to prelimbic (PL) projection inhibits cue-induced reinstatement

**Figure 3:**
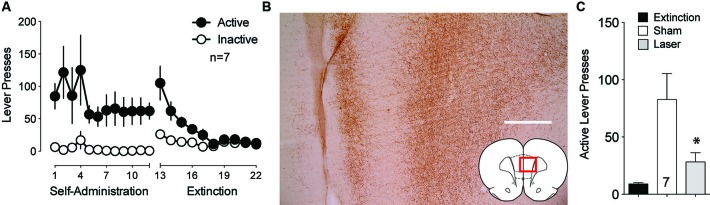
**Optical inhibition of BLA-to-PL fibers blocks cue-primed reinstatement. (A)** Active lever pressing during self-administration and extinction training (*n* = 7). **(B)** ArchT-expressing fibers in the PL after virus injection in the BLA. Scale bar, 300 μm. **(C)** Inactivation of ArchT-expressing fibers in the PL significantly reduces cue-primed reinstatement of cocaine seeking, using a paired Student’s *t*-test with probability values adjusted for multiple comparisons according to Bonferroni, Sham *t*(6) = 3.31, *p* = 0.032; Laser *t*(6) = 2.47, *p* = 0.096. *p < 0.05 comparing laser and sham treatments, *t*(6) = 2.95, *p* = 0.051.

The BLA also sends strong projections to the PL that transfer reward-related information (Fuchs et al., 2007; Mashhoon et al., 2010). Given the importance of the PL to NAcore projection in the relapse to drug seeking (Goldstein and Volkow, 2002; McFarland et al., 2003; Kalivas et al., 2005; Stefanik et al., 2013b), BLA neurotransmission in PL could regulate reinstated cocaine seeking. To test the involvement of the projection from the BLA to the PL in cue-induced reinstatement, ArchT was microinjected into the BLA and optic fibers were implanted in the PL. Figure 3A shows the self-administration and extinction data for these animals. Strong virus expression was detected from BLA injections at the terminal fields in the PL (Figure 3B). Optical inhibition of this pathway attenuated cue-induced cocaine seeking in laser treated animals, but not in sham animals (Figure 3C).

### Histology

**Figure 4:**
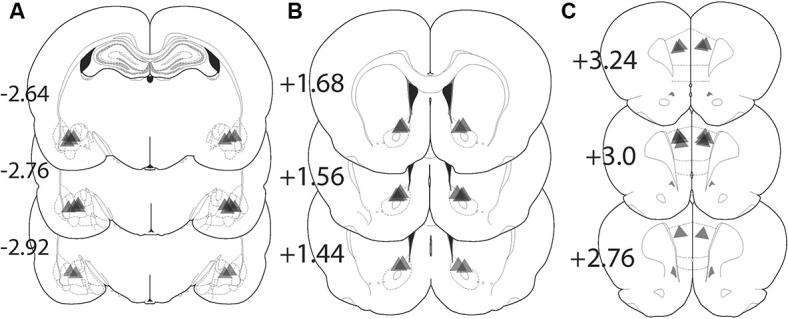
**Location of fiber optic terminations in the BLA, NAcore, and PL for each experiment**. Triangle shape shows the approximate predicted perimeter of light diffusion in the brain. The apex of the triangle corresponds with the histologically identified ventral termination of the fiber optic and the triangle illustrates the approximate size of the illuminated tissue. **(A)** Locations of fiber terminations in the BLA following BLA virus injection. **(B)** Locations of fiber optic termination in the NAcore following BLA virus injection. **(C)** Fiber optic terminations in the PL following BLA virus injection. The numbers refer to the location of the coronal section in millimeters relative to bregma.

Figure 4 shows the location of the fiber optic termination in each experiment. The area of illumination was estimated from the tip of the histologically identified fiber optic tip to expand in a cone shape for 0.5 mm in length and diameter from the most ventral penetration (Yizhar et al., 2011). While virus spread can be seen in a more widely distributed area of the amygdala, the placement of fiber implantations in the BLA were tightly focused just dorsal to the structure and medial to the external capsule and allow for precise targeting of the BLA alone (Figure 4A). Animals with fiber placement outside of the histologically identified BLA were excluded from analysis. Fiber implantations in the NAcore were located primarily dorsal to the anterior commissure (Figure 4B). Figure 4C shows the location fiber optic implants in the PL located lateral to the midline.

## Discussion

The BLA is a key neural structure involved in cue-induced cocaine seeking (Buffalari and See, 2010). Projections from the BLA to the NAc have also been implicated in these behaviors. As previous studies have relied primarily on unilateral pharmacological inactivation to demonstrate the role of the projection, they suffer from a potential interpretational inaccuracy since inactivating projections from the BLA to other regions that influence drug seeking behavior that in turn project to the NAc could be mediating the behavioral inhibition. The PL is one region that might be indirectly influenced since pharmacological and optogentic inhibition of the PL to NAc show that this pathway is critically involved in cocaine reinstatement (McFarland et al., 2003; Stefanik et al., 2013b). Additional evidence also suggests that the BLA-to-PL projection is critical to cue-induced reward seeking (Fuchs et al., 2007; Mashhoon et al., 2010). To address this issue, we used the inhibitory optogenetic construct ArchT to selectively inactivate the BLA or its projections terminating in the NAc or PL. We demonstrate that inhibition of the BLA or either projection is sufficient to markedly reduce cue-induced cocaine seeking. Together, these findings suggest a more nuanced influence of BLA projections on NAc function that previously thought. Thus, while BLA neurotransmission directly to the NAc provides necessary information to facilitate cue-induced cocaine seeking, the BLA can indirectly influence NAc function via selective activation of PL projections to the NAcore.

A role for activation of the PL to initiate behavior may seem paradoxical in the context of a recent finding by Chen et al. (2013) who were able to suppress compulsive cocaine seeking by optogenetically stimulating hypofunctioning PL neurons during the seeking phase of a cocaine-seeking task. They theorize that chronic drug use induces a hypofunctional state in the PFC causing habitual behavior to supersede cognitive control, and thereby promoting compulsive drug seeking. In line with this view, the hypofunctioning PFC and compulsive drug seeking observed by Chen et al. (2013) were reduced by enhancing activity in the PL. Conversely, the results of this and previous work (McFarland et al., 2003; Stefanik et al., 2013b) support a view that PFC activity is needed in order to recognize and integrate information from a number of structures to initiate drug-seeking behavior. Although seemingly paradoxical in the context of drug seeking paradigms, these studies are consistent with a well-established role for the PFC as a site where computations and decisions are made to execute adaptive responses that can include both behavioral activation and inhibition (Ghazizadeh et al., 2012).

The BLA conveys information to the PL about the cues that have been previously paired with reward delivery (Fuchs et al., 2007; McGinty and Grace, 2008; Mashhoon et al., 2010). Interestingly, while the glutamatergic projection from the BLA has been shown to excite PL neurons (Little and Carter, 2013), a proportion of the BLA projections inhibit pyramidal cell firing (Floresco and Tse, 2007) by synapsing onto interneurons in the PL (Dilgen et al., 2013), providing a potentially paradoxical feed forward inhibition of neurotransmission. BLA activation of both pyramidal neurons and interneurons might cause selective attention for cocaine-associated cues while simultaneously suppressing responses for non-drug stimuli. Consistent with this hypothesis, stimulation of BLA neurons can excite or inhibit PL pyramidal neurons projecting to the NAc (McGinty and Grace, 2008, 2009a,b). Work in other brain regions also supports the idea of selective suppression of information may work to funnel information into specific downstream circuits (Liang et al., 2013). Selective input from the BLA onto interneurons in the PL could be serving to gate other information arriving into the PL that might compete with the drug-paired stimuli. Coupled with direct excitation of NAc medium spiny neurons by BLA afferents, the NAc can then process a multi-layered association between the environmental stimulus and a given behavior. Given this possibility, future studies need to address the relationship between BLA-to-PL neurotransmission on selective populations of PL neurons projecting to NAc during the processing of drug-associated cues.

## Author contributions

Michael T. Stefanik and Peter W. Kalivas were responsible for study concept and design. Michael T. Stefanik conducted the behavioral studies and histology. Michael T. Stefanik and Peter W. Kalivas wrote the manuscript.

## Conflict of interest statement

The authors declare that the research was conducted in the absence of any commercial or financial relationships that could be construed as a potential conflict of interest.
